# Sinus node dysfunction in patients with Fontan circulation: could heart rate variability be a predictor for pacemaker implantation?

**DOI:** 10.1007/s00246-019-02092-5

**Published:** 2019-03-27

**Authors:** Jenny Alenius Dahlqvist, Urban Wiklund, Marcus Karlsson, Katarina Hanséus, Eva Strömvall-Larsson, Anders Nygren, Håkan Eliasson, Annika Rydberg

**Affiliations:** 1 Department of Clinical Sciences, Pediatrics, Umeå University, 901 85 Umeå, Sweden; 2 Department of Radiation Sciences, Biomedical Engineering, Umeå University, Umeå, Sweden; 3 Department of Clinical Sciences Lund, Children Heart Centre, Skåne University Hospital, Lund University, Lund, Sweden; 4 Department of Cardiology, The Queen Silvia Children’s Hospital, Sahlgrenska University Hospital, Institute of Clinical Sciences, Gothenburg University, Gothenburg, Sweden; 5 Department of Women’s and Children’s Health, Karolinska Institutet, Karolinska University Hospital, Stockholm, Sweden

**Keywords:** Congenital heart disease, Fontan circulation, Pacemaker, Sinus node dysfunction, Heart rate variability, Poincaré analysis

## Abstract

Sinus node dysfunction (SND) causes significant morbidity in patients after Fontan surgery. Heart rate variability (HRV) reflects the autonomic regulation of the heart, and changes in HRV have been associated with SND in adults. We aimed to study whether changes in HRV could be detected in 24-h electrocardiographic (ECG) recordings in Fontan patients with SND. We compared HRV results from two patient groups; patients with Fontan circulation who later required a pacemaker due to severe SND (*n* = 12) and patients with Fontan circulation and SND, without indication for pacemaker treatment (*n* = 11), with two control groups; patients with Fontan circulation without SND (*n* = 90) and healthy controls (*n* = 66). The Poincaré plot index SD2 (representing changes in heart rate over 24-h) and the very low-frequency (VLF) HRV component were significantly higher in both SND groups, both compared with healthy controls and patients with Fontan circulation without SND. In SND patients with pacemakers, SD2 and VLF were slightly reduced compared to SND patients without pacemaker (*p* = 0.06). In conclusion, in Fontan patients with SND the HRV is significantly higher compared to healthy controls and Fontan patients without SND. However, in patients with severe SND requiring pacemaker, SD2 and VLF tended to be lower than in patients with SND without pacemaker, which could indicate a reduced diurnal HRV in addition to the severe bradycardia. This is a small study, but our results indicate that HRV analysis might be a useful method in the follow-up of Fontan patients regarding development of SND.

**Electronic supplementary material:**

The online version of this article (10.1007/s00246-019-02092-5) contains supplementary material, which is available to authorized users.

## Introduction

The Fontan surgical procedure was first described for tricuspid atresia in 1971 and is currently used for palliation in a wide spectrum of patients with univentricular heart defects [1]. The outcome of patients with Fontan circulation has improved over time due to the development of diagnostics, cardiac surgery, and intensive care procedures.

Arrhythmias of various kinds remain one of the major problems after Fontan surgery [2, 3]. Sinus node dysfunction (SND) is found in 11–45% of patients with Fontan circulation [4, 5, 18, 23] and is one of the major indications for pacemakers in this group [6, 7]. Pacemaker treatment in patients with SND has a favorable effect on hemodynamics, may relieve symptoms such as fatigue, and permits effective treatment with anti-arrhythmic drugs [6].

Heart rate variability (HRV) has been shown to be a marker of autonomic nervous system regulation of the heart. Changes in HRV are also associated with arrhythmias such as supraventricular and ventricular arrhythmias [8, 9]. Specifically, in studies by Bergfeldt et al. and Sosnowski et al., HRV was described as significantly higher in adult patients with SND than in controls [10, 11]. Patients with SND had an abnormal pattern on Poincaré plots for HRV-analysis [10]. Furthermore, in adults, measures of HRV were profoundly predictive of high-degree atrioventricular (AV) block after acute myocardial infarction [12], and abnormal HRV patterns were associated with a higher risk of mortality [13].

In the pediatric population, HRV was reduced in patients with Fontan circulation [14, 15]. Changes in HRV may also present in Fontan patients who develop supraventricular tachycardia, even before the onset of arrhythmia [16]. However, whether HRV can predict the need for pacemaker treatment, in children with Fontan circulation, has not been studied before.

Our aim in this study was to determine whether changes in HRV could be detected in 24-h electrocardiogram (ECG) recordings in patients with Fontan circulation and SND. Therefore, we compared HRV in four different groups; patients with Fontan circulation who later developed the need for a pacemaker due to severe SND; patients with Fontan circulation and SND but without indication for pacemaker treatment; patients with Fontan circulation without SND; and healthy controls.

## Materials and methods

### Patients and controls

To identify all Swedish patients with Fontan circulation who were treated with pacemaker, we searched four registers: SWEDCON (The Swedish Registry of Congenital Heart Disease), the Swedish ICD and pacemaker registry, and registries at the two Swedish centers for pediatric cardiac surgery; Queen Silvia Children’s Hospital in Gothenburg and Skåne University Hospital in Lund. After identification of the patients with Fontan circulation, the patients’ diagnoses were confirmed at each hospital. Informed consent was obtained from the patients or their legal guardians. This study was approved by the Regional Ethical Review Board in Umeå.

In total, 599 patients were identified. Of these, 78 (78/599 = 13%) had a pacemaker. The median follow-up time after Fontan surgery was 11.2 (range 0.02–35.1) years.

Among patients with pacemaker treatment, six patients were deceased. Seventy-two patients or their legal guardians were contacted and 58 gave written informed consent to a retrospective study of the patient’s 24-h ECG recordings and medical records. A group of patients with Fontan circulation without a pacemaker (101 whereof 11 with SND) in whom also legal guardians had given consent to study of medical records and 24-h-ECG recordings, were also included.

The healthy control group consisted of 66 children and youths (33 girls and 33 boys) who underwent a 24-h ECG recording at a median age of 9.7 (range 1.1–17.6) years as previously described [15]. The healthy controls underwent echocardiography and 24-h ECG, with normal findings.

### Methods

Medical records were reviewed for date of birth, gender, anatomical diagnoses, type and date of surgical interventions, late complications, medication at the time the 24-h ECG and data from echocardiographic reports. Semiquantitatively assessed ventricular function by echocardiography was graded on a scale from I to IV, where I was evaluated as poor and IV as good. AV-valve regurgitation was graded on a scale from 0 to 4 with 0 as no regurgitation and 4 as a large regurgitation.

To perform HRV analyses, we contacted each hospital and asked for data from Holter recordings (24-h ECG) made prior to pacemaker implantation. All recordings were edited according to standard procedures for arrhythmia analyses of Holter recordings, including the detection of abnormal beats such as extra systolic beats and disturbances. The annotated and edited RR tables were exported from the Holter system and used in the HRV analyses.

SND was defined as one or more of the following; (1) minimal or mean heart rate 2 SD below the mean value for age and gender [17], (2) predominant junctional rhythm, (3) sinus pauses of three or more seconds on Holter recording and/or (4) HR peak during exercise lower than 80% of the predicted value for age and gender [3, 18].

### HRV analysis

HRV in the 24-h ECG recordings was analyzed using Poincaré plots and by power spectrum analysis. In the Poincaré analysis, each RR interval was plotted as a function of the previous RR interval as a scatter plot. HRV was quantified by the standard deviation (SD) along the line of identity (SD2), representing changes in mean RR, and by the SD along the line that is perpendicular to the line of identity (SD1), representing the magnitude of the beat-to-beat variability [19].

The power spectrum analysis of the beat-to-beat fluctuations in RR intervals was performed by means of fast Fourier transformation as described previously [20]. Spectral power (P) was determined in three frequency regions of interest: very low-frequency region (VLF: 0.003–0.04 Hz), low-frequency region (LF: 0.04–0.15 Hz), and high-frequency region (HF: 0.15–0.50 Hz). HF represents mainly the parasympathetic component, and LF represents both the sympathetic and parasympathetic components of the cardiac autonomic modulation. The total power was also determined in the frequencies from 0.003 to 0.50 Hz. Finally, the LF/HF ratio was calculated. All spectral indices were calculated as average data over the complete recording period (up to 24 h).

### Statistics

Data were presented as frequencies, means with SD, or medians and ranges.

HRV results were presented as *Z*-scores based on the age-dependency in controls, which were modeled as a quadratic regression line. Thus, a *Z*-score of 0 is equivalent to the mean for controls of the same age, and a *Z*-score of + 1 is equivalent to one SD above the mean of the controls. Elliptic confidence intervals were constructed using principal component analysis of the bivariate distribution of *Z*-scores for controls and patients with total cavopulmonary connection (TCPC) without SND, respectively. Further information concerning the mathematical formulas for age-dependency and *Z*-score calculation are presented in the Online Appendix.

*Z*-scores for the four groups, healthy controls, patients with Fontan circulation without SND, patients with Fontan circulation with SND without pacemaker treatment (TCPC/SND) and patients with Fontan circulation with a pacemaker implantation because of SND (TCPC/SND/PM), were compared using analysis of variance, where post hoc tests were performed using group-wise *t* tests.

Statistical significance was defined as *p* < 0.05. All data and statistical analyses were performed with MATLAB R2017b (Mathworks Inc, Natick, MN, USA) and IBM SPSS Statistics for Windows, version 24.0 (IBM Corp. Armonk, NY, USA).

## Results

### 24-h ECG recordings

One hundred-and-one patients with Fontan circulation had previously undergone 24-h ECG monitoring. Amongst them, 11 patients with Fontan circulation, without subsequent pacemaker implantation, fulfilled one or more of the criteria for SND on the 24-h recording. In the group of Fontan patients with a pacemaker, retrospective data were collected from a total of 61 pre-pacemaker 24-h ECG recordings in 19 patients. In 39 patients, pre-pacemaker 24-h-ECG recordings were not available. Forty-two 24-h ECG recordings from before pacemaker implantation were available from 12 patients, with SND as the main indication for pacemaker implantation. HRV analysis was performed on the latest recording before pacemaker implantation.

### Clinical characteristics of Fontan patients without SND

Of the 90 patients with TCPC, but no SND, 27 (30%) were girls. Forty-three patients (48%) had a left, and 40 patients (44%) had a right morphology of their systemic ventricle. Five patients (6%) had both a left and a right ventricular morphology. Data were missing for two (2%) patients. Median age at completion of TCPC was 2.5 (0.8-7.0) years. Concerning the type of TCPC, 42 patients (47%) had lateral tunnel (LT) and 48 (53%) had extracardiac conduit (EC). During follow-up after 24-h ECG recording, two patients had died. Ventricular function, assessed by echocardiography, was graded on a scale from I-IV; 70 patients (78%) had good ventricular function (grade IV) and 17 patients (19%) had grade III ventricular function. In three patients (3%), data were missing. Regarding AV regurgitation, 29 patients (32%) had no regurgitation, 39 (43%) had grade 1 regurgitation, 16 patients (18%) had grade 2 regurgitation, and two patients (2%) had grade 3 regurgitation. Echocardiographic data were missing in four (4%) cases. At the time of the 24-h ECG registration, three patients were treated with digoxin. None of the patients were treated with a beta-blocker or other anti-arrhythmic drugs, and 14 (16%) were treated with an angiotensin-converting enzyme (ACE) inhibitor.

### Clinical characteristics of TCPC patients with SND but without pacemaker treatment

Eleven patients, six girls and five boys fulfilled the criteria for SND on 24-h ECG. Six patients had a left and five patients had a right morphology of their systemic ventricle. Median age at completion of TCPC was 1.8 (1.3–4.0) years. Seven patients had LT, and four had EC. None of the patients had a history of protein-losing enteropathy, thrombosis, or plastic bronchitis. No patient had a heart transplant, one patient died during follow-up. All had grade III–IV/IV ventricular function assessed with echocardiography. Regarding AV regurgitation, four patients had no regurgitation, six had grade 1 regurgitation and one patient had grade 2 regurgitation. At the time of the 24-h ECG registration, none of the patients were treated with a beta-blocker or other anti-arrhythmic drugs.

### Clinical characteristics in patients with Fontan circulation and pacemaker treatment because of SND

Data concerning the group of 12 patients with SND (eight female and four male) and at least one 24-h ECG recorded before pacemaker-implantation are presented in Table 1. Median time from 24-h ECG analysis to pacemaker implantation was 1.2 years (2 days to 4.8 years). On echocardiography at the time of the 24-h ECG recording, all patients had good ventricular function (grade III-IV/IV). Only one patient had AV valve regurgitation of grade 2/4; in the other patients, AV-valve regurgitation was assessed as 0–1/4. None of the patients had a history of protein-losing enteropathy, thrombosis, or plastic bronchitis. No patient had a heart transplant or died during follow-up.

**Table 1:** Clinical characteristics of TCPC patients with pacemaker treatment because of SND

Pat no	Diagnosis	Neonatal surgeries	Age of TCPC (years)	Type of TCPC	Indication for pacemaker implantation		Medication at Holter	Age pacemaker (years)
					ECG-findings	Symptoms		
1	Left isomerism DORV with LV hypoplasia, MA	Norwood + Sano shunt	2.4	EC	Bradycardia	Not known	Aspirin	2.5
2	AVSD with LV hypoplasia	BT	3.2	EC	Bradycardia, frequent episodes of nodal rhythm, SVES in couplets	Fatigue	Propranolol	3.2
3	TA, VSD, PS	BT, Norwood	4.1	EC	Nodal rhythm	Not known	0	4.2
4	AS with LV hypoplasia	BT, DKS	1.9	LT	Bradycardia, episodes of nodal rhythm	Fatigue, dizziness	Aspirin Furosemide, Spironolactone	6.3
5	RV hypoplasia, multiple large VSD	PAB	4.0	EC	Tachy-brady	Fatigue	Sotalol	6.6
6	DORV with LV hypoplasia, MA	PAB	3.6	EC	Bradycardia, Bradycardia	Fatigue	0	7.2
7	TA, VSD	BT	1.7	LT	Bradycardia	Fatigue	Aspirin	8.5
8	AS with LV hypoplasia, VSD	BT, CoA: end-to-end, DKS	1.8	EC	Bradycardia, sinus pause > 3 s	Fatigue	Aspirin	8.7
9	TA, TGA	PAB, DKS	1.6	LT	Bradycardia	Syncope	Warfarin	9.9
10	MA, TGA, PS, VSD	BT	1.8	LT	Bradycardia	Fatigue	Warfarin	10.1
11	DILV, TGA, VSD	BT, DKS	2.6	LT	Episodes of nodal rhythm and of AV-block II	Fatigue	Aspirin Captopril	14.1
12	DILV, TGA, VSD, CoA	PAB, CoA op	1.7	LT	Tachy-brady, bradycardia	Fatigue	Aspirin Furosemide. enalapril	16.1
Mean			**2.6**					8.1

*DORV* double outlet right ventricle, *LV* left ventricle, *RV* right ventricle, *MA* mitral atresia, *AVSD* atrioventricular septal defect, *TA* tricuspid atresia, *VSD* ventricular septal defect, *PS* pulmonary stenosis, *AS* aortic stenosis, *TGA* transposition of the great arteries, *DILV* double inlet left ventricle, *CoA* coarctation of the aorta, *BT* Blalock-Taussig shunt, *DKS* Damus–Kaye–Stansel, *PAB* pulmonary artery banding, *EC* extracardiac conduit, *LT* lateral tunnel

### Analysis of HRV

#### HRV in patients with TCPC without SND

**Fig. 1:**
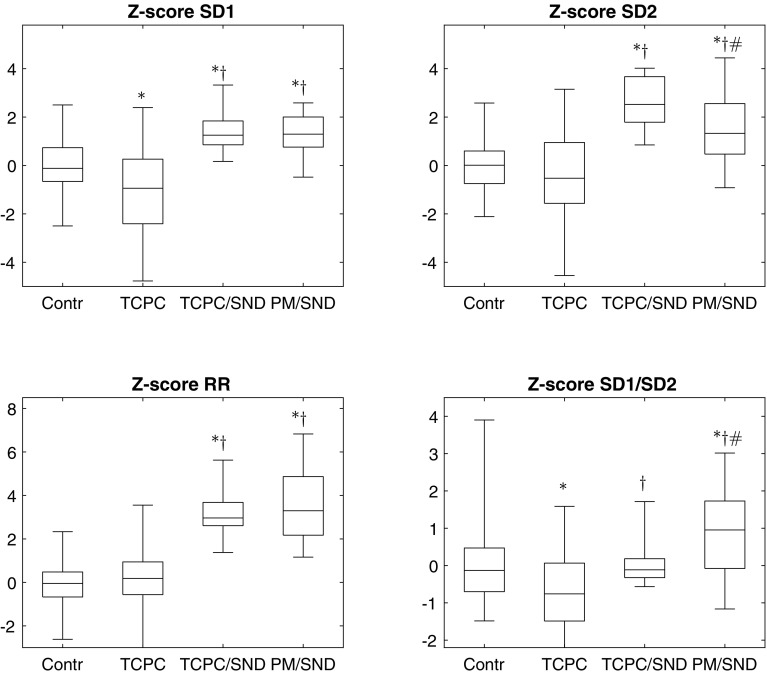
Boxplot of the Z-scores of the Poincaré indices. Boxes represent the median and interquartile range, while the whiskers show the range. Statistically significant differences were derived from analysis of variance and pairwise post hoc tests. *p < 0.05 versus controls. ^†^*p* < 0.05 versus TCPC without SND. ^#^*p* = 0.06 TCPC/SND versus TCPC/SND/PM. *TCPC* total cavopulmonary connection, *SND* sinus node dysfunction, *PM* pacemaker. SD1 and SD2 were log-transformed and age-corrected based on data from controls before *Z*-score calculation

Compared to healthy controls, patients with TCPC without SND had significantly lower HRV parameters: SD1, SD1/SD2, *P*_tot_, *P*_VLF_, *P*_LF_, and *P*_HF_ (Table 2; Fig. 1).

**Table 2:** Values are *Z*-scores derived from age-dependency in controls

	TCPC no SND (*n* = 90)	TCPC/SND no PM (*n* = 11)	TCPC PM/SND (*n* = 12)	ANOVA *p* value
RR interval	0.20 (1.14)	3.11 (1.12)^*,†^	3.49 (1.76)^*,†^	< *0.001*
SD1	− 1.05 (1.67)*	1.34 (0.91)^*,†^	1.27 (0.96)^*,†^	< 0.001
SD2	− 0.34 (1.67)	2.62 (1.11)^*,†^	1.53 (1.57)^*,†,#^	< 0.001
SD1/SD2	−0.68 (0.95)*	0.04 (0.63)†	0.82 (1.25)^*,†,#^	< 0.001
P_tot_	−1.39 (2.17)*	1.80 (0.94)^*,†^	0.87 (1.13)^†^	< 0.001
P_VLF_	−1.09 (2.26)*	2.55 (0.98)^*,†^	1.11(1.38)^*,†,#^	< 0.001
P_LF_	−1.81 (2.67)*	1.59 (1.01)^†^	0.88 (1.39)^†^	< 0.001
P_HF_	−1.48 (1.91)*	0.88 (0.97)^†^	0.45 (0.99)^†^	< 0.001
P_LF_/P_HF_	0.29 (0.93)	− 0.10 (0.99)	− 0.10 (1.01)	0.18

*p* values were derived from analysis of variance (ANOVA) post hoc tests

${P_{tot}}$ total power, *P*_*VLF*_ power of very low frequency component, *P*_*LF*_ power of low frequency component, *P*_*HF*_ power of high frequency component, *SD1 and SD2* Poincaré plot measures (see text for explanation), *TCPC* total cavopulmonary connection, *SND* sinus node dysfunction, *PM* pacemaker

*p < 0.05 versus controls

^†^*p* < 0.05 versus TCPC without SND

^#^*p* = 0.06 TCPC/SND versus TCPC/SND/PM

#### HRV in Fontan patients with SND but without pacemaker treatment (TCPC/SND)

All HRV indices (except for the ratios SD1/SD2 and *P*_LF_/*P*_HF_) were significantly higher in the *TCPC*/*SND* group compared with healthy controls. Moreover, all HRV indices were significantly higher in the *TCPC*/*SND* group than in patients with TCPC without SND. (Table 2; Fig. 1).

#### HRV in Fontan patients before pacemaker due to SND (TCPC/SND/PM)

All Poincaré indices and P_VLF_ were significantly higher in the TCPC/SND/PM group compared with healthy controls. Moreover, all HRV indices were significantly higher in the TCPC/SND/PM group than in patients with TCPC without SND. Two indices, SD2 and P_VLF_, and the ratio SD1/SD2 were slightly reduced in TCPC/SND/PM group compared to the TCPC/SND group (*p* = 0.06) (Table 2; Fig. 1).

**Fig. 2:**
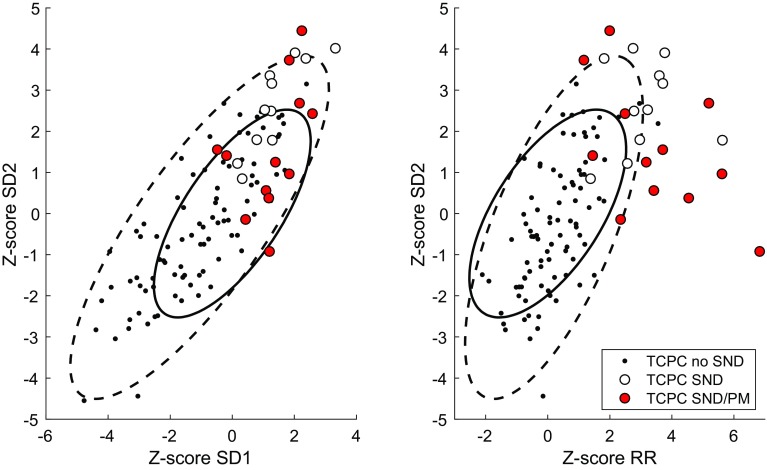
The relationship between Z-scores for RR and SD2. Ellipses show 95% confidence intervals based on a principal component analysis of data from controls (thick solid lines) and from TCPC patients without SND (dashed lines), respectively. *TCPC* total cavopulmonary connection, *SND* sinus node dysfunction, *PM* pacemaker

**Fig. 3:**
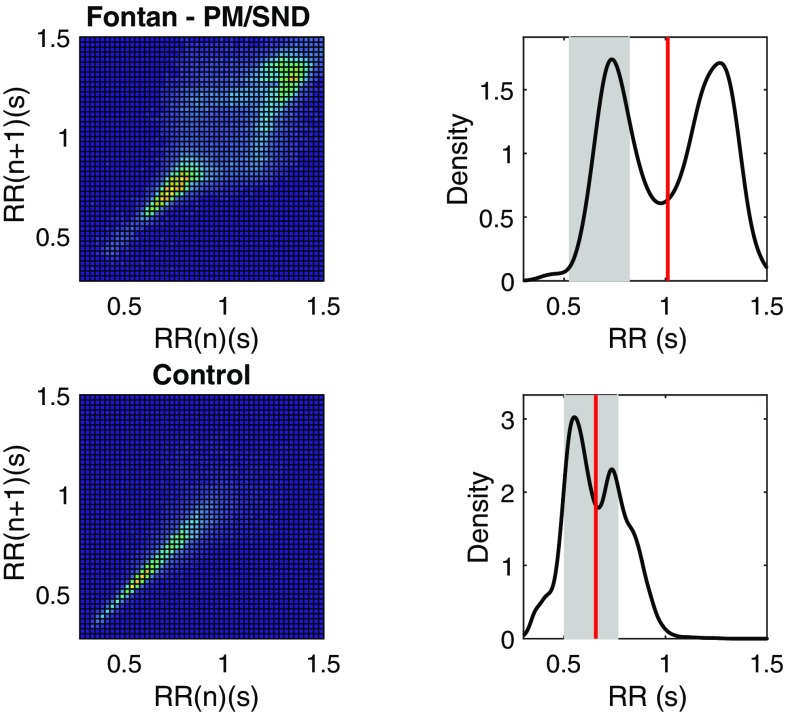
Examples of Poincaré (left) and density (right) plots of RR intervals for two subjects. The upper panels show plots from an 8-year-old girl with Fontan circulation, who later received a pacemaker because of symptomatic SND. The graph has a rhomboid shape due to changes between nodal and sinus rhythm. The lower panels show a recording from a healthy child of the same age. The Poincaré plots are color coded; bright color corresponds to regions with the highest number of values, which also corresponds to the location of peaks in the density plots. Density plots: grey area corresponds to the 95% CI for the expected mean RR at the age of the patient (see Fig. 2 in [17]). Vertical line is the mean RR for the patient

Figure 2 shows how the Poincaré index, SD2 was associated with mean *R*–*R* interval. The figure shows the bivariate 95% confidence intervals for controls (solid line) and TCPC patients without SND (dashed line). Note that a high SD2 reflects a large variation and a low SD2 corresponds to a low variation in mean heart rate over the 24-h recording period. As shown by the solid ellipsis, 10/11 TCPC/SND patients and 11/12 TCPC/SND/PM patients had deviating and different patterns of RR and SD2 compared to controls, where the majority of TCPC/SND patients also had a deviating pattern compared with the TCPC patients without SND. Furthermore, six in the TCPC/SND/PM group with high *Z*-scores for RR also had a deviating pattern compared to the TCPC/SND group. These six TCPC/SND/PM patients had relatively normal SD2, which reflects that they had relatively moderate changes around the markedly prolonged mean RR; thus, they presented with the most pronounced bradycardia. Two in the TCPC/SND/PM group presented with high SD2, but relatively normal mean RR. As shown in the left panel of Fig. 2, there were few TCPC/SND and TCPC/SND/PM patients that presented with a deviating pattern when compared to the corresponding bivariate confidence intervals for SD1 and SD2. Figure 3 shows two examples of how the RR interval changed over 24 h: as a Poincaré plot showing the relationship between one RR interval and the next; and as a Density plot showing the overall distribution of RR intervals.

## Discussion

The main finding of this study is that patients with Fontan circulation with SND have HRV changes in 24-h ECG recordings showing significantly higher HRV than both healthy controls and patients with Fontan circulation without SND. This is, to the best of our knowledge, the first study on HRV in pediatric patients with Fontan circulation with SND.

In patients with Fontan circulation with a fixed stroke volume, cardiac output will be limited by pronounced bradycardia. In junctional escape rhythms, with loss of atrioventricular synchrony, ventricular filling is reduced, which also results in decreased stroke volume [21, 22]. SND is a common, late complication post-Fontan surgery [18, 23]. Patients with Fontan circulation reportedly experience a reduced quality of life, compared to healthy controls [24], with arrhythmia and chronotropic incompetence shown to be predictive of the level of health-related quality of life impairment in these patients [25, 26].

Pacemaker treatment in patients with SND is indicated when the patient has symptoms related to bradycardia, provided that other causes have been excluded [27]. In a study of 116 patients with TCPC, Bossers et al. reported that SND was found in 33 patients (29%), only three of them met indications for a pacemaker implantation [3]. Close surveillance is important because SND can become symptomatic over time. Moreover, SND in patients with Fontan circulation is associated with the development of atrial flutter [28]. Thus, a method for predicting progressive SND requiring pacemaker treatment would be helpful to clinicians caring for these patients.

We found reduced HRV as analyzed by spectral analysis (*P*_tot_, *P*_VLF_, *P*_LF_, and *P*_HF_) among TCPC-operated patients with no pacemaker compared to healthy controls. This has previously been shown by us and others [14, 15, 20], and HRV has been shown to be successively reduced over time after TCPC surgery [29]. Surgery on the caval veins of the right atrium may affect the intracardiac ganglia located at the cavo-atrial junctions [14]. HRV is influenced by the autonomic nervous system, and reduced HRV reflects decreased parasympathetic and increased sympathetic activity. From the adult setting, we know that HRV is reduced in cases of ventricular dysfunction due to sympathetic overdrive [12]. However, heart failure alone is not likely to explain the reduced HRV in this setting since the majority of Fontan patients were assessed with echocardiography which showed good ventricular function (grade III-IV/IV) and minimal AV-regurgitation (grade 0–1/4).

In this study, Fontan patients with SND had a significantly elevated HRV compared with both healthy controls and Fontan patients without SND. It is important to note that elevated HRV can be due to normal cardiac autonomic modulation with parasympathetic predominance, but also reflects the presence of abnormal heart rate patterns such as heart rate modulations due to cardiac arrhythmias and conduction disturbances. In adults without congenital heart disease, HRV has been shown to be significantly higher in patients with SND than in healthy controls [10, 11]. Using Poincaré plot analysis, these patients with SND showed abnormal patterns [10]. In agreement with the findings in the adult population, we found abnormal patterns of Poincaré plots in this pediatric population with SND (Fig. 3). In the Poincaré plot, high SD2 reflects diurnal variation of the heart rate and a low SD2 is seen where there is reduced variability over 24 h. Although group means of SD1 and SD2 were higher in patients with pacemaker treatment because of SND than in patients without SND, SD2 in the Poincaré analysis tended to decrease in patients who needed pacemaker treatment (*p* = 0.06). This tendency of reduction in HRV in patients with SND, who develop need for pacemaker treatment, was also seen in the spectral analysis (*P*_VLF_, *p* = 0.06).

It is important to relate SD2 to the mean RR interval as shown in Fig. 2. Using this bivariate graphical analysis of RR and SD2, HRV changes in patients with severe SND and a need for pacemaker treatment can be visualized. The differences found between Fontan patients with SND with and without pacemaker suggest that patients with SND start with an elevated SD2 due to the presence of episodes of bradycardia, either because of intermittent episodes or due to bradycardia during the night. At this point, the mean heart rate is lower than in healthy controls, but the heart rate can still rise. This could result in an increased SD2. Possibly, with progression of SND, the patient’s heart rate fails to increase. At this point, the SD2 will decrease and appear to be normal, whereas mean heart rate will further decrease. This could result in false normal Poincaré plot presentation despite more severe SND. HRV analysis in two of the patients with SND who subsequently had a pacemaker implantation, showed high SD2 but relatively normal mean RR, which could reflect the presence of episodes of pronounced bradycardia in between times with more normal heart rates.

High SD1 reflects a high beat-to-beat variability, but could also reflect the presence of frequent large changes in mean heart rate. Thus, in the case of SND, an increased SD1 could be explained by repeated shifts in the heart rhythm between sinus rhythm and junctional rhythms. Another possibility is the presence of other forms of arrhythmia such as atrial extra-systole or atrioventricular conduction disturbances that would also contribute to increased beat-to-beat variability.

### Limitations

This is a retrospective study. Unfortunately, permanent storage of data from 24-h ECG recordings is not routine at all Swedish hospitals. Therefore, Holter data were not saved for more than 12 Fontan patients who subsequently had a permanent pacemaker implanted because of SND. However, despite the small number of patients, the results from the HRV analysis are noteworthy.

Two of the patients in the group of patients with pacemaker treatment because of SND in our study were on beta-blockers at the time of HRV analysis; however, this probably had little impact on our results since beta-blockers are known to have only a modest effect on HRV [27, 30]. Two patients in the pacemaker group had left isomerism, a condition where a normal sinus node is absent, which means the mechanism behind development of SND likely was different to the other types of anatomical malformations. In two cases, the only 24-h ECG recordings stored were made only two days prior to pacemaker implantation. Therefore, the analysis was repeated without these subjects. The results of significant differences in HRV parameters remained.

## Conclusions

In this population of patients with TCPC and SND, 24-h ECG recordings showed higher HRV than both other TCPC patients without SND and healthy controls. Moreover, patients requiring a pacemaker due to SND had pre-implantation 24-h ECG recordings showing a tendency toward decreased HRV compared to patients with TCPC and SND without pacemaker treatment. This indicates that HRV analysis might be useful in the follow-up of Fontan patients regarding the development of more severe SND with need for pacemaker treatment.

## Electronic supplementary material

Below is the link to the electronic supplementary material.

Supplementary material 1 (DOCX 15 KB)
