# Supplementary material for: Sinus node dysfunction in patients with Fontan circulation: could heart rate variability be a predictor for pacemaker implantation?
Source: Pediatr Cardiol. 2019 Mar 27;40(4):685–93. doi: 10.1007/s00246-019-02092-5 (PMC6451711; doi:10.1007/s00246-019-02092-5)
Supplement: Supplementary file 1 — Supplementary material 1 (DOCX 15 KB) [file 246_2019_2092_MOESM1_ESM.docx]

**Supplementary material/ Appendix**

Z-scores were calculated based on age-corrected reference values for controls as:

$$Zscore=\frac{(observed value in a subject-reference value at the age of the subject)}{standard deviation of values for controls around the reference line}$$

The reference values were modelled according to the following second order function for all HRV variables where a significant age-dependency was found,

$\hat{X}_{age}=\beta_{0}+\beta_{1}\cdot age+\beta_{2}\cdot{age}^{2}$

where $age$ is expressed in years. Next, the standard deviation of the age-corrected values for controls was calculated, given by the standard deviation of the differences between observed values, and values predicted by the regression equation:

${SD}_{reference}=SD\left( X_{observed,controls}-\hat{X}_{age, controls} \right)$

Finally, Z-scores for all subjects were calculated as:

$Zscore=(X_{observed}-\hat{X}_{age})/{SD}_{reference}$

The following equations were determined for the Poincaré plot indices

${RR}_{age}=487.0+25.9\cdot age-0.408\cdot{age}^{2} ; Z_{RR}=(RR-{RR}_{age})/67.9$

${SD1}_{age}=1.211+0.076\cdot age-0.003\cdot{age}^{2}; Z_{SD1}=(SD1-{SD1}_{age})/0.171$

${SD2}_{age}=1.992+0.056\cdot age-0.002\cdot{age}^{2}; Z_{SD2}=(SD2-{SD2}_{age})/45.6$

where $RR, SD1$ and $SD2$ are given in ms and the latter two were log10-transformed. No significant age-dependency was found for the ratio $SD1/SD2$. Therefore, this variable was only mean-adjusted before z-score calculation by the equation:

$Z_{SD1/SD2}=(SD12-0.191)/0.063$
